# Development of a Novel Cyclodextrin–Chitosan Polymer for an Efficient Removal of Pharmaceutical Contaminants in Aqueous Solution

**DOI:** 10.3390/ma17143594

**Published:** 2024-07-21

**Authors:** Fadila Oughlis-Hammache, Mohamed Skiba, Lamia Moulahcene, Nicolas Milon, Frédéric Bounoure, Malika Lahiani-Skiba

**Affiliations:** 1 UNIROUEN, Galenic Pharmaceutical Laboratory, NorDIC Inserm U1239, UFR Medicine and Pharmacy, Rouen University, Normandie Univ, 22 Bd Gambetta, F-76183 Rouen, France; 2 Laboratory of Membrane Processes and of Separation and Recovery Techniques, Faculty of Technology, Abderrahmane Mira University, Route de Targua Ouzemmour, Bejaia 06000, Algeria; 3 Faculty of Science and Applied Sciences, Department of Process Engineering, Akli Mohand Oulhadj University, Bouira 10000, Algeria; 4 Institute of Technology, Department of Process Engineering, Akli Mohand Oulhadj University, Bouira 10000, Algeria

**Keywords:** cyclodextrin polymer, chitosan, pharmaceutical residue, adsorption, ibuprofen, progesterone

## Abstract

A novel polymer synthesized by grafting three cyclodextrins onto chitosan was characterized and evaluated for its potential to adsorb two pharmaceutical residues: ibuprofen and progesterone. The influence of various operational parameters, including contact time, initial molecule concentration, pH, ionic strength, and temperature, was investigated. The synthesized polymer exhibits an amorphous and porous structure with a remarkable swelling capacity of 9.5 mmol/g. It demonstrates remarkable adsorption capacities for progesterone and ibuprofen, reaching 90% and 75%, respectively. Kinetic studies reveal that the adsorption of both molecules follows a pseudo-second-order model. A DSC analysis elucidated the adsorption mechanism, which is governed by the formation of inclusion complexes and electrostatic interactions within the polymer network. The polymer’s regeneration after 23 cycles demonstrates its sustainable adsorption efficiency. The combination of chitosan with three cyclodextrins opens up promising new avenues for water treatment and the removal of specific pollutants. This approach significantly improves the material’s selectivity towards target pollutants, offering a significant advantage in pollution remediation applications.

## 1. Introduction

The search for novel high-performance and specific materials has intensified in recent years, driven by the need for enhanced efficiency and selectivity. These materials have often been the subject of research in various fields, particularly in the environment. The growing presence of pharmaceutical residues, a class of emerging environmental contaminants [1,2], has raised concerns due to their potential toxicity to both ecological and human health. These residues have been the focus of numerous scientific investigations.

Progesterone and ibuprofen, widely consumed drugs worldwide, are often found in surface water, municipal and hospital waste, and effluents. 

Among the different methods used in the elimination of pharmaceutical residues [3,4,5,6,7,8,9], the adsorption process is one of the most attractive methods due to its efficiency, ease of implementation, and the availability of different adsorbents [10,11,12]. 

Biopolymers are considered innovative materials that can be modified physicochemically to improve their properties for specific applications. 

Chitosan, a biopolymer derived from chitin, exhibits remarkable adsorption properties due to its numerous amino (–NH_2_) and hydroxyl (–OH) groups that act as active adsorption sites [13,14,15]. However, chitosan has limitations, such as its lack of selectivity, low mechanical strength, and solubility in acidic media, for practical application towards specific compounds [16]. To overcome these limitations, modifications of the functional groups of chitosan by crosslinking and grafting, the generation of nanoparticles and hydrogels, and the association of chitosan with other materials have been developed [17,18,19,20,21].

The grafting of cyclodextrins (CDs), cyclic oligosaccharides derived from starch, onto chitosan is a promising approach to improve its properties [18]. Insoluble CD polymers prepared by crosslinking with crosslinking agents such as glutaraldehyde, epichlorohydrin (Ep), HMDI, and citric acid [22,23,24] find many applications [25,26,27,28].

This study aims to develop a new bio-adsorbent material based on chitosan and three types of CDs (-α, -β, and -ɣ) crosslinked with citric acid for the selective removal of pharmaceutical pollutants in aqueous solution.

After characterization, the adsorption performance of the polymer was evaluated in the removal of two pharmaceutical contaminants, ibuprofen and progesterone, alone and in mixture. The effects of contact time, flow rate, pH of the solution, ionic strength, and temperature on the adsorption rate are studied.

Thermodynamic and kinetic studies were carried out to determine the nature of the adsorption reaction and the appropriate kinetic model. A thermogravimetric analysis (TGA) was undertaken to elucidate the mechanism of the adsorption of pollutants by the synthesized polymer.

The results of this research will contribute to the valorization of chitosan and the development of new solutions for water decontamination.

## 2. Materials and Methods

### 2.1. Materials

#### 2.1.1. Chemicals

The progesterone and ibuprofen were obtained from UP John Company (Hastings, MI, USA) and Hubei Granules-biocause pharmaceutical Co., Ltd. (Wuhan, China), respectively, and were used without further purification.

The chitosan with molecular weight of 300 Da was obtained from SIGMA-ALDRICH and was put to use without further purification. 

The insoluble alpha-beta-gamma-cyclodextrin polymer was procured from start-up, In-Cyclo^®^, Rouen, France.

All other reagents were of analytical grade.

#### 2.1.2. Apparatus

An XRD analysis of the cyclodextrin–chitosan polymer was carried out with a diffractometer (kind Oxpert pro panalytical). The conditions for current and voltage were set at 20 mA and 40 kV, respectively. This analysis utilized a monochromatized X-ray beam from Cu Ka radiation with a wavelength of λ = 0.154 nm. The scan was performed over the range of 3–90° 2θ at a rate of 4° min^−1^.

Scanning electron microscopy (SEM) using a Jeol JSM-6031by with LFD mode was employed to investigate the morphology of the material.

A differential scanning calorimetry (DSC) analysis of ibuprofen, progesterone, and the cyclodextrin–chitosan polymer before and after adsorption was performed with a PERKIN ELMER instrument. Appropriate amounts of the samples were placed in perforated aluminum pans and were heated from 35 to 300 °C at a scanning rate of 10 °C/min, under a nitrogen purge gas flow of 25 mL/min.

A JASCO UV/VIS spectrophotometer was used to quantify progesterone and ibuprofen in aqueous solutions. Their respective maximum absorption wavelengths are 250 nm and 223 nm.

Size exclusion chromatography coupled with multi-angle laser light scattering (SEC-MALS) was employed to determine the molecular weight and the average molar mass. 

pH measurements are made using a digital pH meter model, Inolab.

### 2.2. Methods

#### 2.2.1. Synthesis of Cyclodextrin–Chitosan Polymer

**Figure 1:**
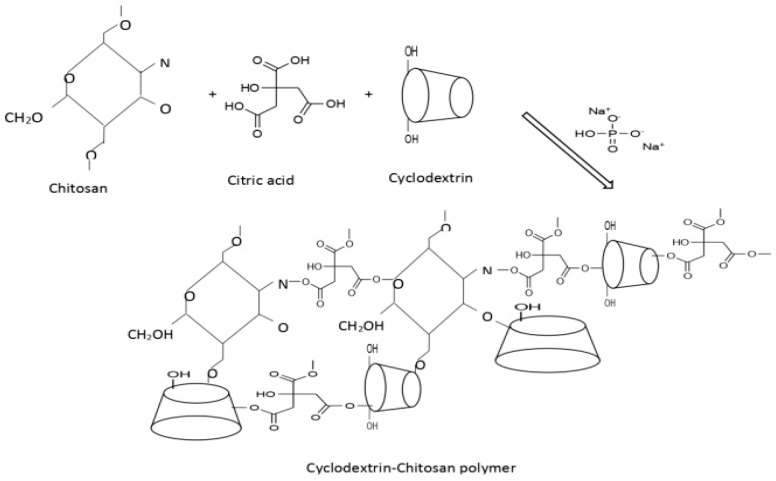
Crosslinking of cyclodextrin–chitosan polymer.

The insoluble tetrapolymers (α-, β-, γ-), cyclodextrins–chitosan, were synthesized by a direct melt polycondensation with grafted chitosan (Figure 1): Initially, 3 g of citric acid and 0.3 g of chitosan were introduced into a reactor maintained at a temperature of 140 °C. Then, a mixture of 1 g of α-cyclodextrin, 1.3 g of β-cyclodextrin, 1.5 g of γ-cyclodextrin, and 1 g of sodium phosphate dibasic (Na_2_HPO_4_) as a catalyst was added. The solution was stirred under vacuum for 30 min. The solid residue, obtained in accord with the invention [24,26], was washed successively with 20 mL of water three times.

#### 2.2.2. Swelling Capacity of the Cyclodextrin–Chitosan Polymer

To determine the swelling capacity (SC) of this material, a sufficient amount of it is immersed in water, which is then removed at different times and wiped until it has a constant weight [27]. 

The swelling capacity (%) is defined as the mass lost before and after swelling, and it was determined as follows [28]:

$$\mathrm{SC}=\frac{We-W0}{W0}\;(\%)$$

where W0 and W_e_ are the mass of sample at dry state before and after swelling equilibrium (g), respectively.

#### 2.2.3. Total Acidic Groups of the Cyclodextrin–Chitosan Polymer

The estimate of total acidic groups (TA), as well as ester and carboxylic groups, was carried out by a titration method. A total of 0.1 g of the polymer was introduced in a beaker, which contained 20 mL of 0.1 M of NaOH solution, the mixture was then blended for 15 h at 30 °C. The cyclodextrin–chitosan polymer was dissolved and hydrolyzed.

The titration of the final solution is carried out using a 0.1 M HCl solution until obtaining pH 7 [22]. The following equation was used to determine the TA:

$$TA=\frac{C(V_{0}-V_{1})}{W}$$

C: the concentration of HCl solution (mmol L^−1^); 

V_0_: volume of HCl solution consumed by blank solution (L); 

V_1_: volume of HCl solution consumed by sample solution (L); 

W: weight of the cyclodextrin–chitosan polymer (g).

#### 2.2.4. Determination of Molar Mass of Cyclodextrin–Chitosane Polymer 

The molar mass (Mn) of the cyclodextrin–chitosan polymer is determined by SEC/MALS. This method allows the determination of mass distributions of polymers. Size Exclusion Chromatography (SEC) is used to separate macromolecules according to their size (their hydrodynamic volume in solution). For that, the polymer solutions are injected and then eluted onto columns filled with non-adsorbent porous material. At the outlet of the column, the fractions are separated according to their characteristics. Contrary to the techniques based on standard polymers and a simple detection of concentrations (usually with a differential refractometer), the addition of a second detection by diffusion of multiangle laser light, sensitive to molecular weights, gives access to instantaneous variations of the gyration radius and to the average molar mass (Mw) of the eluted species at each time of elution. 

### 2.3. Adsorption Experiments 

**Figure 2:**
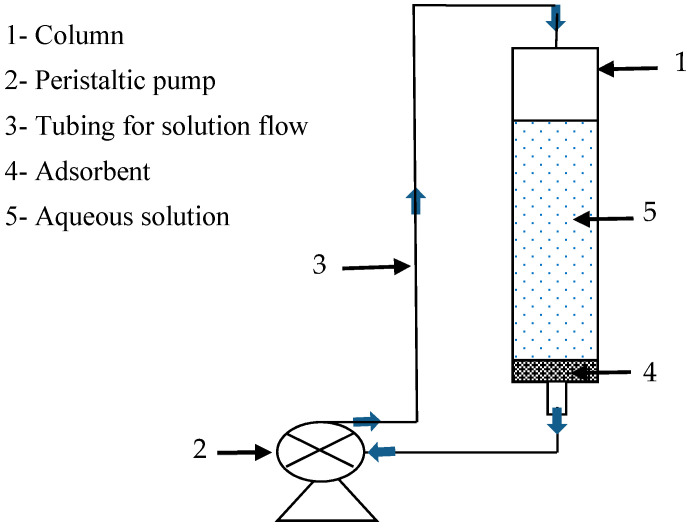
Experimental setup for pharmaceutical removal.

Adsorption experiments were performed using a fixed-bed column (inner diameter 35 mm, height 120 mm) containing varying amounts of adsorbent material. The column was filled with 60 mL of the solution to be treated, ensuring adequate contact time for efficient adsorption (Figure 2). A peristaltic pump circulated the solution in a closed loop through the adsorbent in a downward direction at a constant, predetermined flow rate. Samples were collected at regular intervals using a pipette during the solution percolation process for subsequent analysis.

Equilibrium times were determined by conducting experiments for different durations: 6 h for ibuprofen and 2 h for progesterone. Each experiment was repeated three times to ensure reproducibility of the results. Stock solutions of ibuprofen (30 mg/L) and progesterone (20 mg/L) were prepared in deionized water and a deionized water/ethanol mixture 60/40 (*v*/*v*), respectively. Solutions at different concentrations were obtained by diluting the stock solutions.

The adsorption kinetics were followed at regular time intervals. Solution samples of 800 µL were taken and analyzed by UV-Vis spectrometry at specific wavelengths: 223 nm for ibuprofen and 250 nm for progesterone. The amount of solute adsorbed by the insoluble cyclodextrin–chitosan polymer (qt, mg/g) and the removal efficiency (%) were calculated using the following equations:

(1) $$q_{t}=\frac{V(C_{0}-C_{t})}{m}$$

(2) $$\mathrm{Removal}\;(\%)=\frac{{(C}_{0}-C_{t})}{C_{0}}\;*100$$

C_0_ (mg/L): initial concentration of pharmaceuticals;

C_t_ (mg/L): concentration of pharmaceuticals at time t;

V (L): volume of solution;

m (g): mass of cyclodextrin–chitosan polymer.

## 3. Results 

### 3.1. Characterization of Cyclodextrin–Chitosan Polymers

#### 3.1.1. XRD Structural Analysis

**Figure 3:**
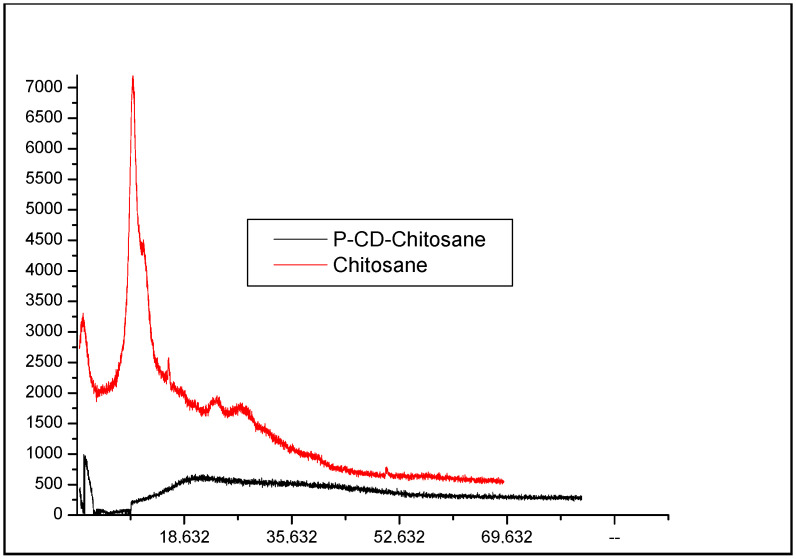
X-ray diffraction pattern of chitosan and cyclodextrin–chitosan polymer (P–CD–chitosan).

Figure 3 presents the X-ray diffraction (XRD) patterns of chitosan and P–CD–chitosan. The XRD pattern of chitosan exhibits an intense peak at 2θ = 10°, characteristic of its semi-crystalline structure. In contrast, the XRD pattern of P–CD–chitosan shows a broad and diffuse peak at 2θ = 20°, accompanied by the absence of distinct diffraction peaks, indicating the amorphous nature of the synthesized polymer. This amorphous structure is attributed to the homogeneous dispersion of cyclodextrin molecules within the chitosan matrix.

#### 3.1.2. Morphology Analysis

**Figure 4:**
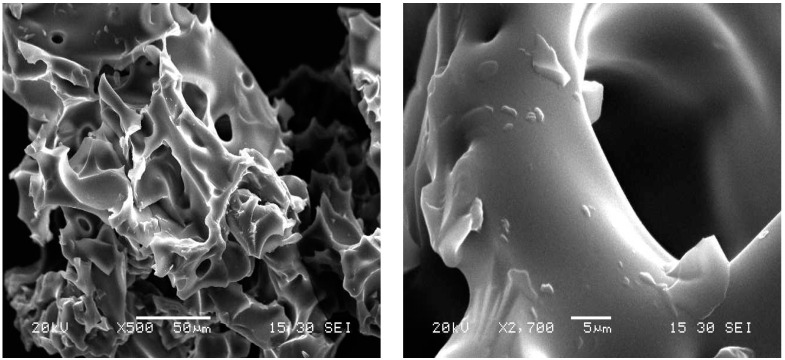
SEM images of cyclodextrin–chitosan polymer with different magnifications.

The scanning electron microscopy (SEM) images (Figure 4) clearly reveal the morphology of the polymer surface in the dry state. The polymer has a sponge structure with thick, homogeneous, and smooth cavities. It has a porous structure with mesopores and some nanocavities. These properties allow it a high swelling capacity (Table 1), which allows for the rapid diffusion of the adsorbates in the polymer matrix [27].

#### 3.1.3. Properties of Cyclodextrin–Chitosan Polymer

The swelling capacity (SC), total acidic (TA), molar mass (Mn) and average molar mass (Mw) results are reported in Table 1. We note a high swelling capacity of the polymer.

### 3.2. Effects of Operating Parameters 

The effects of the operating parameters (contact time, solution flow rate, pH, ionic strength, and temperature) on the adsorption rate of the two pharmaceutical molecules onto the synthesized cyclodextrin–chitosan polymer are investigated in a closed-loop fixed-bed column. The initial concentrations of ibuprofen and progesterone solutions, selected based on their solubility limits, are 10 mg/L and 30 mg/L, respectively. The different measurements were made on solution samples taken at different times.

#### 3.2.1. Contact Time Effect

**Figure 5:**
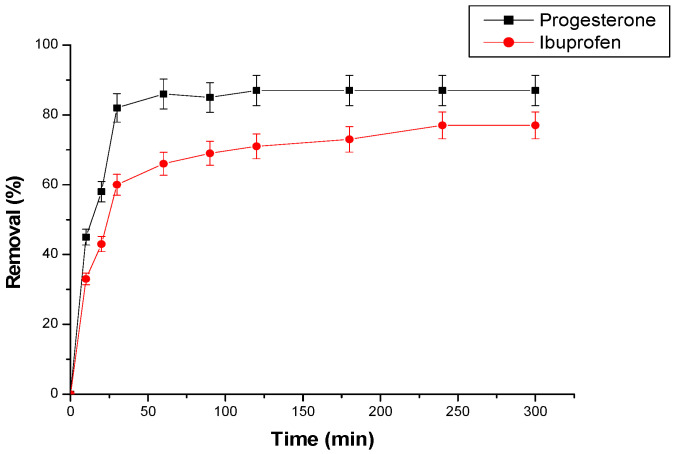
Effect of contact time on the adsorption of pharmaceuticals. Conditions: flow rate (1.5 L/h), temperature 25 °C; initial concentration of progesterone and ibuprofen are, respectively, 10 ppm and 30 ppm; pH = 2 (ibuprofen) and pH = 7 (progesterone).

Samples of the ibuprofen and progesterone solutions are collected from the column and analyzed. Figure 5 shows the adsorption rates of the samples by the cyclodextrin–chitosan polymer over time.

Adsorption yields increase with increasing contact time until an equilibrium is achieved. A rapid increase in the adsorption rate is observed during the first 10 min and then slows down to reach equilibrium after 50 min for progesterone and 250 min for ibuprofen.

#### 3.2.2. Effect of Flow Rate 

**Figure 6:**
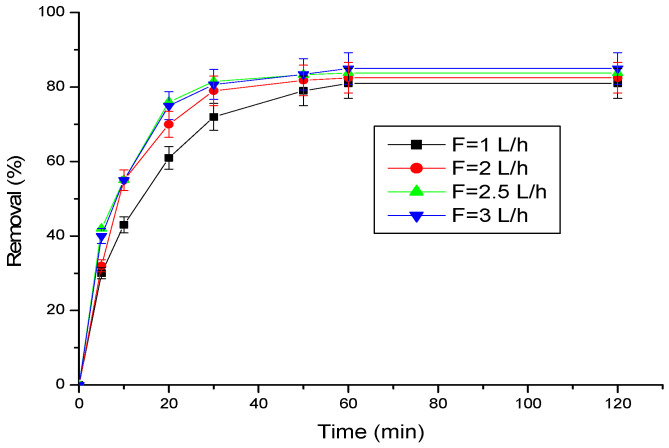
Effect of flow rate (F) on progesterone removal. Conditions: amount of adsorbent (25 mg), temperature (25 °C), initial concentration of progesterone (10 ppm), pH = 7.

Figure 6 depicts the relationship between the flow rate and the progesterone adsorption rate. Higher flow rates enhance progesterone removal, likely due to two mechanisms: increased mass transfer of solute from the bulk solution to the solid surface and a thinning of the boundary layer surrounding the solid with increasing cycles.

#### 3.2.3. Effect of Initial Solution pH

**Figure 7:**
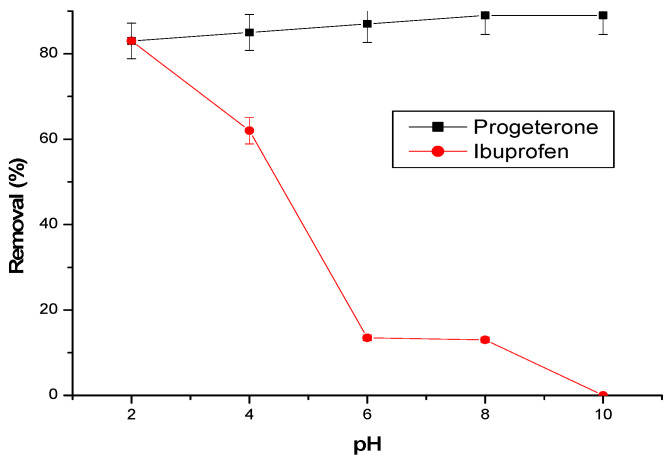
Effect of pH on pharmaceuticals adsorption. Conditions: adsorbent amount = 25 mg; flow rate = 3 L/h; temperature 25 °C; initial concentration of progesterone and ibuprofen are, respectively, 10 ppm and 30 ppm.

The effect of the initial pH is shown in Figure 7. The initial pH significantly influences the adsorption of ibuprofen, but less so the adsorption of progesterone.

The best adsorption efficiency of the two molecules is observed at a pH of 2. However, the elimination of ibuprofen decreases with the increase in the initial pH. For a pH higher than the pKa = 4.91 of ibuprofen [26,29,30], the molecule becomes deprotonated, becomes negatively charged, and, thus, prevents the formation of inclusion complexes with the cyclodextrin cavities. Additionally, there is an electrostatic repulsion with the negative charge of the acidic groups in the cyclodextrin–chitosan polymer formed at basic pH.

In the case of progesterone, the elimination rate remains practically constant (85%) at pH values below the pKa equal to 9.7; progesterone in molecular form has a greater affinity for cyclodextrin by forming inclusion complexes.

The results show that the elimination mechanism of ibuprofen and progesterone is mainly governed by the inclusion of the molecules in the cyclodextrin cavities.

#### 3.2.4. Effect of Ionic Strength

**Figure 8:**
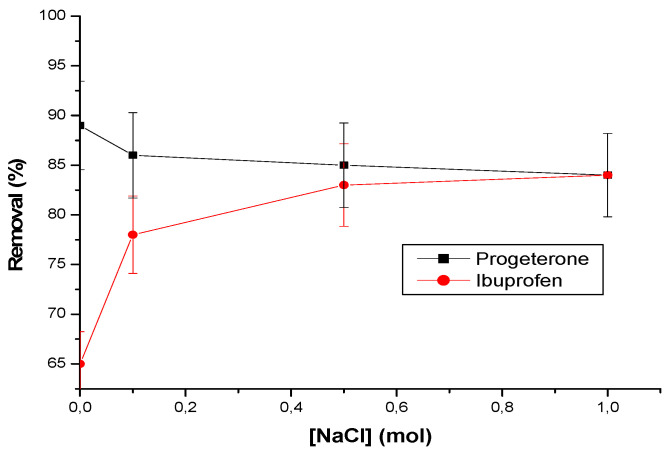
Effect of NaCl concentration on pharmaceuticals removal. Conditions: amount of adsorbent = 25 mg; flow rate = 3 L/h, temperature 25 °C; initial concentration of progesterone and ibuprofen are, respectively, 10 ppm and 30 ppm; pH = 2 in case of ibuprofen and pH = 7 in case of progesterone.

The influence of ionic strength on the adsorption of the two pharmaceutical products is an interesting phenomenon. Figure 8 illustrates these variations.

Progesterone adsorption shows minimal variation with increasing ionic strength. This suggests that progesterone is not strongly influenced by electrostatic interactions linked to ionic strength.

Adsorption of ibuprofen increases with ionic strength. This increase can be attributed to the solubility effect of ibuprofen on its negative charge. In other words, ibuprofen becomes less soluble as ionic strength increases, thus promoting electrostatic interactions.

Additionally, the formation of inclusion complexes with cyclodextrins may also contribute to this increased adsorption.

In summary, solubility and electrostatic interactions play a key role in the adsorption of these pharmaceuticals as a function of ionic strength.

#### 3.2.5. Effect of Temperature 

**Figure 9:**
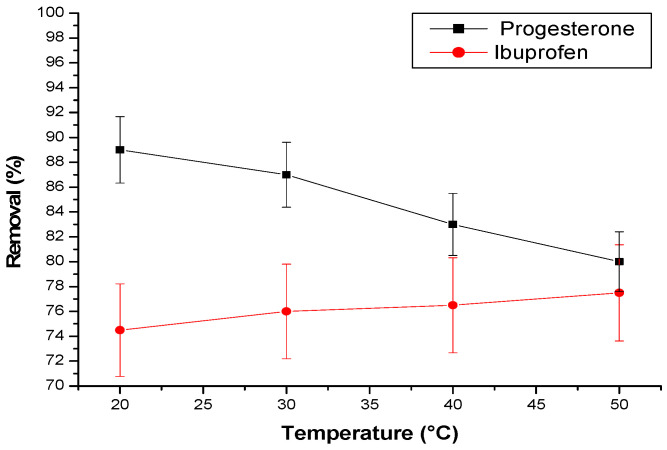
Effect of temperature (T) on pharmaceuticals. Conditions: adsorbent amount is 25 mg; flow rate = 3 L/h; initial concentration of progesterone and ibuprofen are, respectively, 10 ppm and 30 ppm; pH = 2 in case of ibuprofen and pH = 7 in case of progesterone.

The influence of temperature on the elimination of progesterone and ibuprofen using a cyclodextrin–chitosan polymer was thoroughly investigated. The findings reveal a contrasting effect of temperature on the elimination of these two molecules (Figure 9).

For progesterone, a decrease in elimination was observed as the temperature rose. This trend suggests that lower temperatures favor the adsorption of progesterone within the polymer. Conversely, the elimination of ibuprofen follows an inverse trajectory, increasing with temperature.

This differential behavior could be explained by two main mechanisms:

- Polymer pore expansion: At higher temperatures, the polymer pores expand, facilitating the diffusion of ibuprofen molecules into its matrix.
- Creation of new active sites: Elevated temperature could also lead to the creation of new active sites within the polymer, thereby increasing its capacity to adsorb ibuprofen.

In summary, temperature plays a crucial role in the interaction between these substances and the cyclodextrin–chitosan polymer, distinctly influencing their respective elimination.

### 3.3. Thermodynamic Study

Thermodynamic parameters such as entropy (ΔS) and enthalpy (ΔH) are determined from the plot of ln (K_d_) versus temperature (Van’t Hoff Equation (6)).

The free energy change can be obtained by the following formula:

ΔG = ΔH − T. ΔS(3)

The free energy change can also be expressed as follows:

ΔG = ΔG^0^ + RT lnK_d_. (4)

At equilibrium ΔG = 0

ΔG^0^ = −RT lnK_d_

On the other hand,

ΔG^0^ = ΔH^0^ − T. ΔS^0^ (5)

(6) $$\ln K_{d}=\frac{∆S^{0}}{R}-\frac{∆H^{0}}{RT}\;\;\text{(Van’t Hoff law)}$$

**Figure 10:**
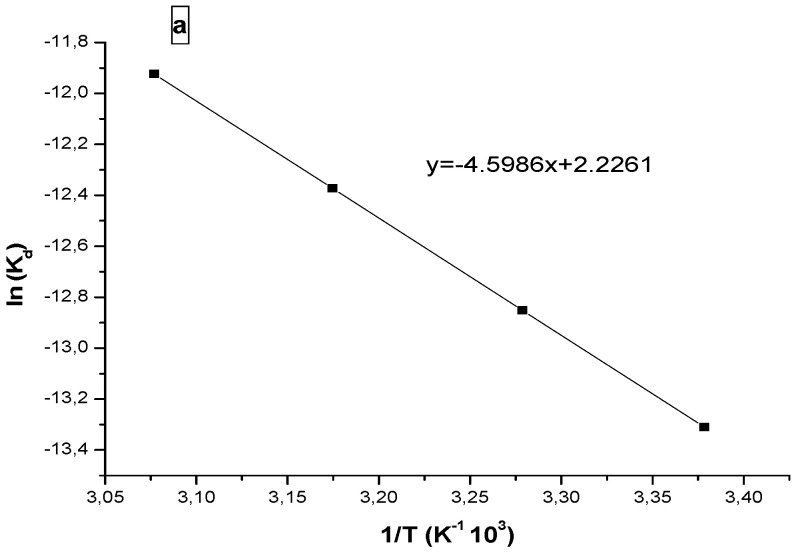

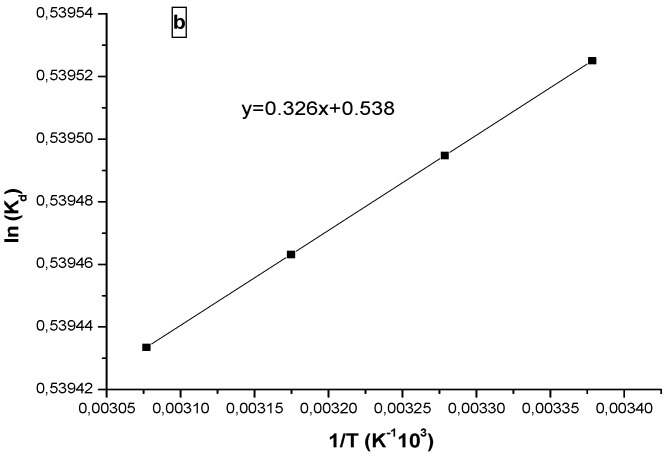
Vant’ Hoff plots of ln(K_d_) versus 1/ T for (**a**) progesterone and (**b**) ibuprofen.

Figure 10 shows the plot of lnKd versus the reciprocal of absolute temperature (T); the thermodynamic parameters are calculated from the slope and the resulting right ordinate. The free energy variation can be obtained by Equation (5).

The thermodynamic parameters calculated are summarized in Table 2. The positive value of enthalpy for progesterone indicates that the adsorption process on the polymer is endothermic. The negative value of the enthalpy in case of ibuprofen indicates an exothermic adsorption process.

The positives values of the entropy for the two pharmaceuticals show that the adsorbed molecules on the polymer surface are organized in a more random fashion compared to those in the aqueous phase [31].

### 3.4. Adsorption Kinetic Modeling 

Adsorption kinetics are of considerable practical interest for the implementation of the adsorbent. It allows us to highlight the specificity of the physicochemical interactions between the solute and the adsorbent and to obtain the adsorption rate and the amount adsorbed at equilibrium.

The adsorption kinetics of ibuprofen and progesterone on the surface of the synthesized polymer are studied at pH = 2 for ibuprofen, pH = 7 for progesterone, and a temperature of 25 °C, using the pseudo-first-order (Equation (7)), pseudo-second-order (Equation (8)), and Elovich (Equation (9)) models. 

The linear form of pseudo-first-order equation is:

ln (q_e_ − q_t_) = ln q_e_ − k_1_ t (7)

k_1_ (min^−1^): pseudo-first-order adsorption rate constant.

q_e_: amount adsorbed per unit mass at equilibrium; 

q_t_: amount adsorbed per unit mass at any time t;

Linear form of pseudo second order equation [29,32]:

(8) $$\frac{t}{q_{t}\;}\;=\frac{1}{k_{2}q_{e}^{2}}+\frac{t}{q_{e}}$$

k_2_ (g/mg.min): pseudo-second-order adsorption rate constant.

The linear form of Elovich equation [31]:

q_t_ = β ln (αβ) + β ln t (9)

α and β are the Elovich coefficients.

The validity of each model could be verified by the linear regression value (correlation coefficient, R^2^) and a normalized standard deviation Δq (%), which can be obtained by the following equation [32]:

(10) $$\Delta q\;(\%)=\sqrt{{\frac{\sum \left[(q_{exp}-q_{cal})/q_{exp}\right]}{n-1}}^{2}}\times 100$$

q_exp_: experimental adsorbed amount per adsorbent mass at equilibrium;

q_cal_: calculated adsorbed amount per adsorbent mass at equilibrium;

n: number of data points.

The R² values obtained from the linear form of the models are mentioned in Table 3 and Table 4.

**Figure 11:**
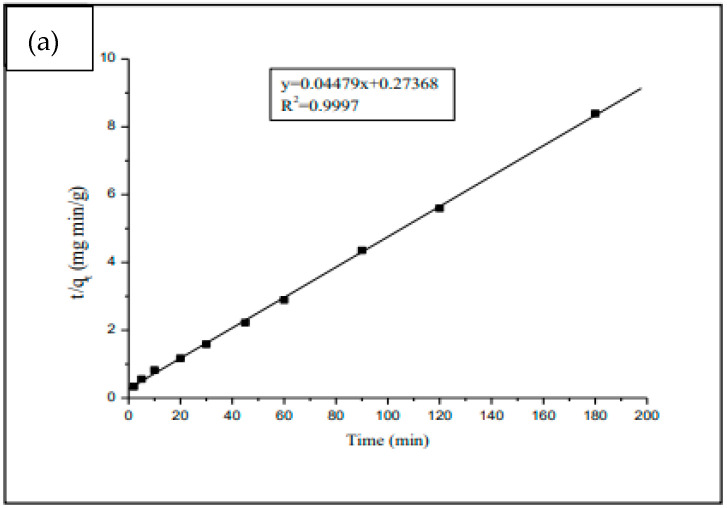

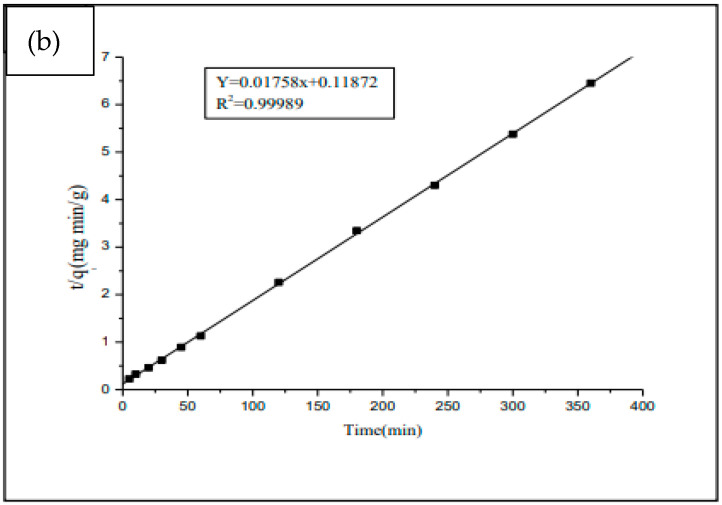
Curve-fitting plot of a pseudo-second-order model for adsorption kinetics of progesterone (**a**) and ibuprofen (**b**), respectively.

Among the three models tested, the pseudo-second-order model appears to be the most favorable in the adsorption process of ibuprofen and progesterone, indicating strong interactions between the studied molecules and the synthesized polymer. Indeed, the values of the regression coefficients R² of the kinetic lines (a1) and (a2) (Figure 11) for progesterone and ibuprofen, respectively, are close to 1. In addition, the low values of the normalized standard deviation ∆q confirm the validity of the model. 

The higher goodness of fitting for the pseudo-second-order model could be ascribed to the nature of the cyclodextrin–chitosan polymer, with multiple adsorption sites, which are responsible for different adsorption steps [33]. This model indicates that the adsorption process depends on the properties of the adsorbent and adsorbate.

### 3.5. Adsorption of Progesterone and Ibuprofen on Cyclodextrin–Chitosan Polymer: Alone and Mixed Systems

**Figure 12:**
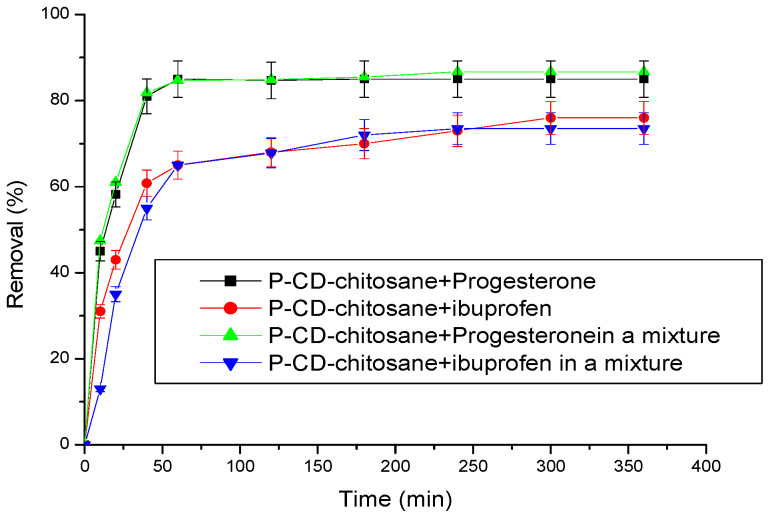
Removal rates of ibuprofen and progesterone alone and in a mixture by the P–CD–chitosan. Conditions: adsorbent mass = 25 mg; flow rate = 3 L/h; temperature 25 °C; initial concentration of progesterone and ibuprofen are, respectively, 10 ppm and 30 ppm; pH = 2.

The adsorption of a mixture containing two molecules onto the cyclodextrin–chitosan polymer was investigated (Figure 12).

Figure 12 shows the remarkable efficacy of the cyclodextrin–chitosan polymer as an adsorbent for progesterone, whether the latter is present alone or in a mixture with another molecule, namely, IB. This outstanding performance can be attributed to the molecular characteristics of progesterone, particularly its shape and size, which favorably predispose it for inclusion within the cavities of gamma CDs through physical interactions. Notably, the adsorption rates observed for progesterone and ibuprofen, alone or in a mixture, remain practically identical, highlighting the polymer’s remarkable ability to maintain its effectiveness even in the presence of another molecule.

### 3.6. Comparative Study of the Adsorption Efficiency of Chitosan, Cyclodextrin Polymer, and Cyclodextrin–Chitosan Polymer 

**Figure 13:**
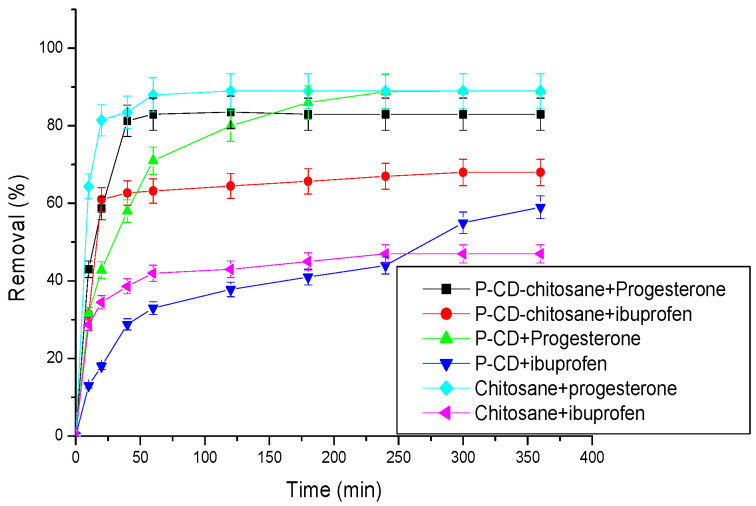
Adsorption rates of ibuprofen and progesterone by the three polymers. Adsorbent mass (25 mg); flow rate (3 L/h); temperature (25 °C); initial concentration of progesterone and ibuprofen are, respectively, 10 ppm and 30 ppm; pH = 2 (ibuprofen) and pH = 7 (progesterone).

This study aims to compare the adsorption efficiency of progesterone and ibuprofen by three polymers: chitosan, (α-, β-, γ-) cyclodextrin polymer, and cyclodextrin–chitosan polymer. Adsorption experiments were carried out using the experimental device illustrated in Figure 2. The results obtained are presented in Figure 13.

- Adsorption of progesterone

Chitosan exhibits the highest progesterone adsorption rates, reaching approximately 90%. Its adsorption kinetics are rapid, with equilibrium reached in about 50 min. This performance can be explained by the porous structure and large specific surface area of chitosan, which favor the physical adsorption of molecules to its surface.

The adsorption kinetics of progesterone on the cyclodextrin polymer are slower. After 200 min, the removal rate reaches 90%. This phenomenon can be explained by the time required for progesterone molecules to find their way into the cavities of the different CDs and form inclusion complexes.

The CDs–chitosan association does not significantly improve progesterone adsorption rates, which remain around 90% after 50 min.

- Adsorption of Ibuprofen

The adsorption rates of ibuprofen by chitosan or by the cyclodextrin polymer are relatively low, reaching about 40%.

The combination of chitosan and CD polymer improves ibuprofen adsorption, with a rate reaching approximately 70%. This improvement can be explained by the creation of a composite material with a larger adsorption surface, favoring specific interactions between the polymers and the ibuprofen molecule.

In conclusion, chitosan proves to be the most effective polymer for progesterone adsorption, while the chitosan–cyclodextrin polymer composite is the most efficient for ibuprofen adsorption. The results of this study highlight the importance of the structure and properties of polymers in the adsorption process.

### 3.7. Physico-Chemical Methods 

#### 3.7.1. X-ray Powder Diffractometry (XRPD)

**Figure 14:**
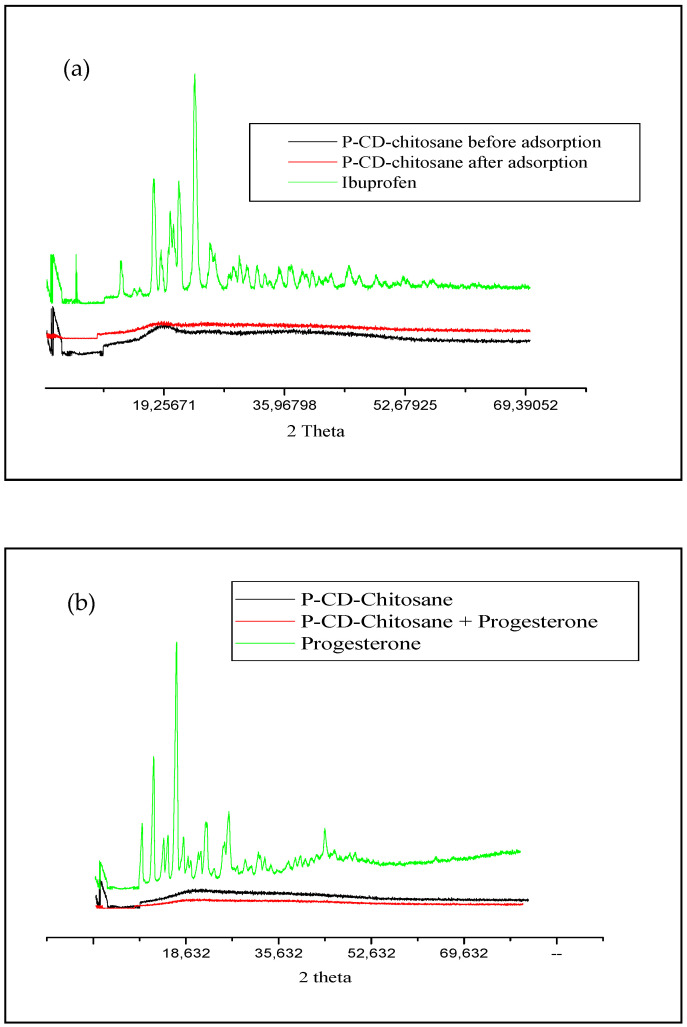
X-ray diffractogram of (**a**) ibuprofen, (**b**) progesterone, and cyclodextrins–chitosan polymers before and after extraction.

The X-ray powder diffractometry (XRPD) analysis revealed an amorphous structure for the cyclodextrins–chitosan polymer, in contrast to the crystalline nature of ibuprofen and progesterone, which exhibited a series of sharp, intense peaks (Figure 14).

Upon extraction of ibuprofen and progesterone, the cyclodextrins–chitosan polymer maintained its amorphous structure, as evidenced by the absence of characteristic diffraction peaks for the drugs. This suggests the formation of a highly disordered inclusion complex between the pharmaceuticals and the polymer matrix, as corroborated by previous studies [34,35]. Alternatively, the lack of drug-related peaks could be attributed to a monomolecular dispersion of ibuprofen and progesterone within the polymeric matrix due to physical interactions [36].

#### 3.7.2. Adsorption Mechanism 

**Figure 15:**
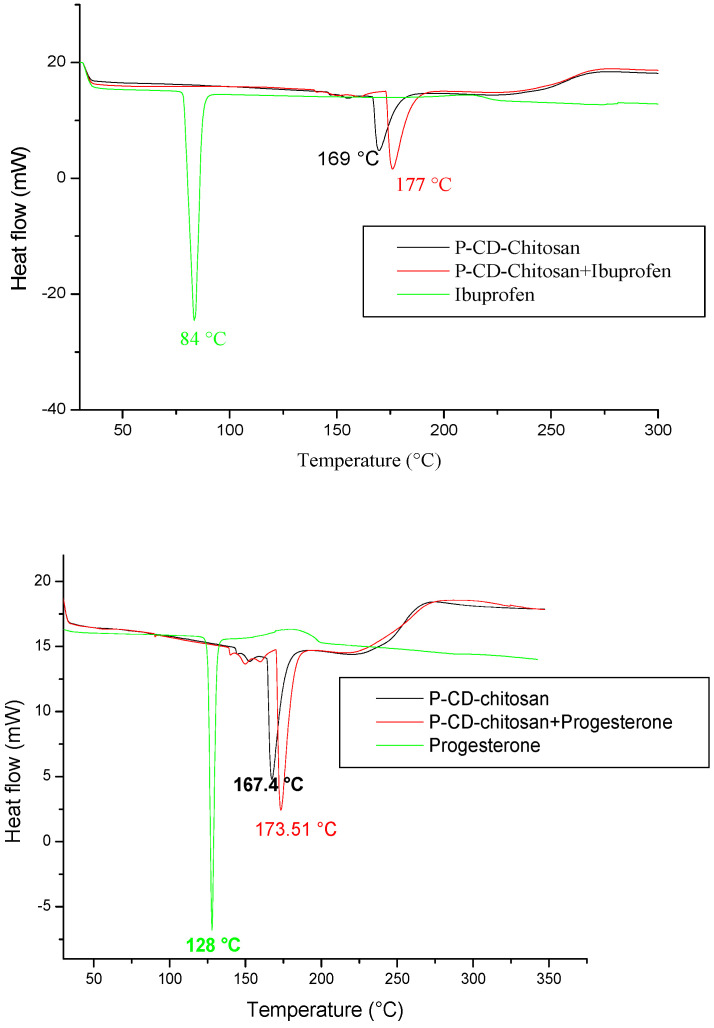
Differential scanning calorimetry (DSC) of ibuprofen, progesterone, and cyclodextrin–tosan polymer before and after extraction of ibuprofen and progesterone.

To elucidate the adsorption mechanism of the synthesized polymer, a differential scanning calorimetric (DSC) analysis of cyclodextrin–chitosan polymer, ibuprofen, and progesterone is performed before and after adsorption (Figure 15).

The thermograms of ibuprofen and progesterone present endothermic peaks at 84 °C and 128 °C, respectively, corresponding to their melting point, while the cyclodextrin–chitosan polymer presents a broad endothermic peak corresponding to the loss of the crystalline water contained in the polymer.

After adsorption, the figure shows a shift and an increase in the amplitude of the endothermic peak of the polymer and the disappearance of the endothermic peaks, corresponding to the melting points of ibuprofen and progesterone. This can be explained by the formation of inclusion complexes under the effect of physical interactions between the molecules and the polymer network.

### 3.8. Regeneration of Cyclodextrin–Chitosan Polymer

**Figure 16:**
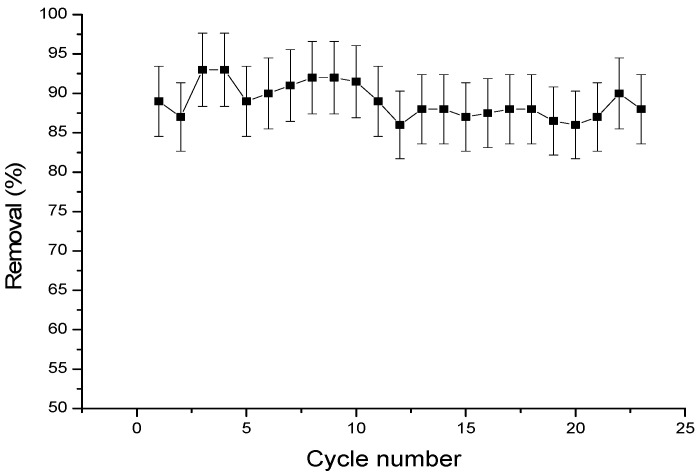
Cycle of adsorption–desorption of progesterone with chitosan–cyclodextrin polymer.

The cyclodextrin–chitosan polymer was regenerated with a water/ethanol mixture in a proportion of (70/30). Figure 16 shows the progesterone elimination rate as a function of the number of regeneration cycles (adsorption/desorption). The adsorption efficiency of the polymer remains constant after twenty-three cycles of use.

## 4. Conclusions 

A novel biopolymer was synthesized by grafting three cyclodextrins (alpha, beta, and gamma) onto chitosan in the presence of a non-toxic cross-linker, citric acid. This polymer was characterized and evaluated for its adsorption and removal potential of two pharmaceutical molecules of different nature and size: ibuprofen and progesterone.

The synthesized polymer exhibits an amorphous and porous structure with a high swelling capacity (71.41%).

Ibuprofen adsorption is strongly influenced by the pH and ionic strength of the solution, while progesterone adsorption is not affected by these parameters.

The DSC analysis indicates that the adsorption of both molecules is mainly due to the formation of inclusion complexes and physical interactions within the polymer network.

The positive entropy values suggest random adsorption of pharmaceutical molecules onto the polymer surface.

Adsorption kinetics follows a pseudo-second-order model for both molecules.

The polymer exhibits a better adsorption capacity for ibuprofen (70%), compared to chitosan (45%) and alpha-, beta-, gamma-cyclodextrin-based polymer (38%).

The progesterone removal percentage reaches 90% with the polymer but does not show a significant improvement compared to chitosan or alpha-, beta-, or gamma-cyclodextrin-based polymer.

The polymer exhibits selective affinity for progesterone, adsorbing it even in the presence of ibuprofen.

The material can be regenerated up to 23 times without significant loss of efficiency, making it a major asset for repeated use.

Grafting cyclodextrins into the chitosan matrix increases the number of adsorption sites and improves the material’s selectivity for specific pharmaceutical molecules.

Its high adsorption capacity, selectivity, and efficient regeneration make this synthesized polymer a promising candidate for large-scale applications in drug delivery and pharmaceutical purification.

Overall, the synthesized biopolymer demonstrates remarkable properties and potential for various pharmaceutical applications.

## Figures and Tables

**Table 1:** Properties of cyclodextrin–chitosan polymer.

SC (%)	TA (mmol/g)	Mn (g/mol)	Mw (g/mol)
71.41	9.50	43,000	310,000

**Table 2:** Thermodynamic parameters of ibuprofen and progesterone adsorption on cyclodextrin–chitosan polymer.

Molecule	C_0_ (mg/L)	T (K°)	ΔH° (J/mol)	ΔS◦ (J/°K.mol)
Progesterone	10	296	38.2319	18.5077
		305		
		315		
		325		
Ibuprofen		296		
	30	305	−2.7103	4.4770
		315		
		325		

**Table 3:** Results of R^2^, k, and Δq (%) for different equations used to model the kinetic adsorption of progesterone by cyclodextrin–chitosan polymer.

Equation	(5)	(6)	(7)
R^2^	0.856	0.9998	0.932
k_1_ (g/mg.min)	-	0.0020	-
Δq (%)	95.08	0.52	9.53

**Table 4:** Results of R^2^, k, and Δq (%) for different equations used to model the kinetic extraction of ibuprofen by cyclodextrin–chitosan polymer.

Equation	(5)	(6)	(7)
R^2^	0.692	0.9997	0.8300
k_2_ (g/mg.min)	-	0.0003	-
Δq (%)	85.87	5.94	8.91

## Data Availability

The data presented in this study are available on request from the corresponding author.
